# Comparative efficacy of ultrasound versus elbow arthrography in treating Monteggia fractures in young children

**DOI:** 10.1051/sicotj/2026078

**Published:** 2026-11-02

**Authors:** YongFei Fan, ChaoYu Liu, XiuLin Ma, WenQiang Xu, HaiYang Yu, Wei Wang

**Affiliations:** Department of Orthopedic Surgery, Fuyang people’s Hospital Affiliated to Anhui Medical University, Anhui Provincial Clinical Research Center for Spinal Deformities, Fuyang People’s Hospital Fuyang Anhui 236000 PR China

**Keywords:** Ultrasound, Monteggia fracture, Young children, Arthrography

## Abstract

*Purpose*: This study aims to compare the effectiveness and clinical value of ultrasound-assisted and arthrography-assisted treatment for acute Monteggia fractures in young children. *Methods*: We conducted a retrospective analysis of 40 cases of acute Monteggia fractures in children treated with Kirschner wire or elastic intramedullary nail fixation between April 2020 and April 2025. Among these cases, 19 were treated under arthrography assistance (Group A), and 21 were treated under ultrasound guidance (Group B). We recorded baseline data for both groups, including gender, age, fracture side and type, associated injuries, time from injury to surgery, internal fixation device, fluoroscopy time, surgical duration, follow-up duration, and internal fixation removal time. Postoperative X-ray examination was performed to assess the quality of fracture reduction. *Results*: Fluoroscopy time and surgical duration in Group B were significantly lower than in Group A (*P* < 0.05). Based on the evaluation criteria for assessing reduction quality, 17 cases were graded excellent, and 2 were considered good in Group A, while in Group B, 20 cases were deemed excellent and 1 was considered good. At the final follow-up, there was no significant difference in the range of elbow flexion, extension, pronation, and supination between the affected limbs of Group A and Group B (*P* > 0.05). *Conclusion*: Ultrasound not only saves surgical time and reduces intraoperative radiation exposure for the patients but also enables the exploration of peripheral nerves, vessels, and soft tissue compressions.

Monteggia fractures are relatively rare, accounting for 1% of elbow fractures in children. Failure to promptly diagnose and administer appropriate treatment may result in forearm rotational dysfunction and limited elbow flexion-extension range of motion [1, 2]. For acute Monteggia fracture, the initial treatment involves manual reduction followed by plaster casting. However, conservative treatment has a failure or redisplacement rate of approximately 20% [3].

For cases of closed reduction failure or difficult-to-reduce acute Monteggia fracture, surgery is the most effective treatment method. However, because of the high proportion of articular cartilage and the incomplete ossification of the radial head epiphysis in young children, conventional radiography cannot visualize these structures, making intraoperative assessment of whether humeroradial joint alignment has been restored challenging [4, 5]. To address this issue, some studies have applied intraoperative arthrography to visualize the structures around the elbow joint, achieving satisfactory treatment outcomes. However, arthrography is associated with soft tissue leakage and an increased risk of elbow joint infection [6].

In recent years, ultrasound has been increasingly used in the intraoperative treatment of elbow fractures in children. As a non-invasive procedure, it not only reduces radiation exposure for patients but also allows for the exploration of blood vessels and nerve injuries around the elbow joint [7]. Therefore, this study aims to compare and analyze the effectiveness and clinical value of ultrasound versus arthrography as adjunctive treatment for acute Monteggia fracture in children.

## Patients and methods

We conducted a retrospective analysis of data from 40 pediatric patients with acute Monteggia fracture who underwent ultrasound-assisted or arthrography-assisted treatment between April 2020 and April 2025. This study was approved by the hospital’s ethics committee, and the guardians of the children authorized the use of their clinical data (No.: [2024]86 Date: 04/22/ 2024). In this study, 19 cases were treated with arthrography assistance (Group A), and 21 cases were treated under ultrasound guidance (Group B). Group A included 11 males and 8 females, with an average age of 5.26 ± 1.73 years (range: 1–7 years). There were 10 left-sided and 9 right-sided fractures, of which one was an open Gustilo I fracture, and two had associated radial nerve injuries. According to the Bado classification, there were 8 cases of Type I, 2 of Type II, 7 of Type III, and 2 of Type IV. A total of 10 males and 11 females were included in Group B, with an average age of 5.05 ± 1.43 years (range: 2–7 years). There were 14 left-sided and 7 right-sided fractures, of which three were open Gustilo I fractures; one had associated radial nerve injuries; and one had distal radius and ulna fractures. According to the Bado classification, there were 9 Type I, 4 Type II, 7 Type III, and 1 Type IV cases.

## Surgical procedure

Both groups of pediatric patients underwent a surgical procedure performed by the same surgical team under general anesthesia. Group A: The status of the ulnar fracture and the position of the radial head were observed under C-arm fluoroscopy. First, restore the length and curvature of the ulna. Depending on the fracture location and type, perform reduction and internal fixation of the ulnar fracture using Kirschner wires or Elastic Stable Intramedullary Nail (ESIN). Then, perform manual reduction of the radial head. Finally, use C-arm fluoroscopy to evaluate the quality of ulnar fixation and the alignment of the radial head. Criteria for successful closed reduction of the humeroradial joint were the humeroradial line passing through the middle one-third of the radial head ossification center on the anteroposterior view and through the center of the capitellum ossification nucleus on the lateral view. Intraoperative elbow joint arthrography was performed to confirm the relationship between the radial head and the distal humerus, using Ioversol as the contrast agent diluted 1:1 with saline. A lateral approach to the elbow joint was taken to inject 0.5–1.0 mL of Ioversol into the joint cavity for arthrography. The elbow joint was gently manipulated to allow even distribution of the contrast agent within the joint (Figure 1). The humeroradial relationship was re-examined under C-arm fluoroscopy in both anteroposterior and lateral views to confirm restoration. Group B: Under ultrasound guidance, exploration of the humeroradial joint and the ulnar fracture site was performed. Under ultrasound guidance, the ulnar fracture was reduced, and fixation was chosen based on the fracture type and location, either through percutaneous pinning or antegrade ESIN. Traction was applied to reduce the radial head. For cases with difficult or failed reduction, ultrasound was used to investigate whether there was soft tissue impingement within the humeroradial joint. If impingement was present, we attempted to use a Kirschner wire to pry and help reduce the impinged soft tissue. After fixation, ultrasound was used again to examine the reduction of the humeroradial joint and ulnar fracture, as well as the stability of the radial head during elbow extension, flexion, and forearm rotation (Figures 2, 3). Finally, fluoroscopy was used to further verify the reduction and internal fixation.

**Figure 1:**
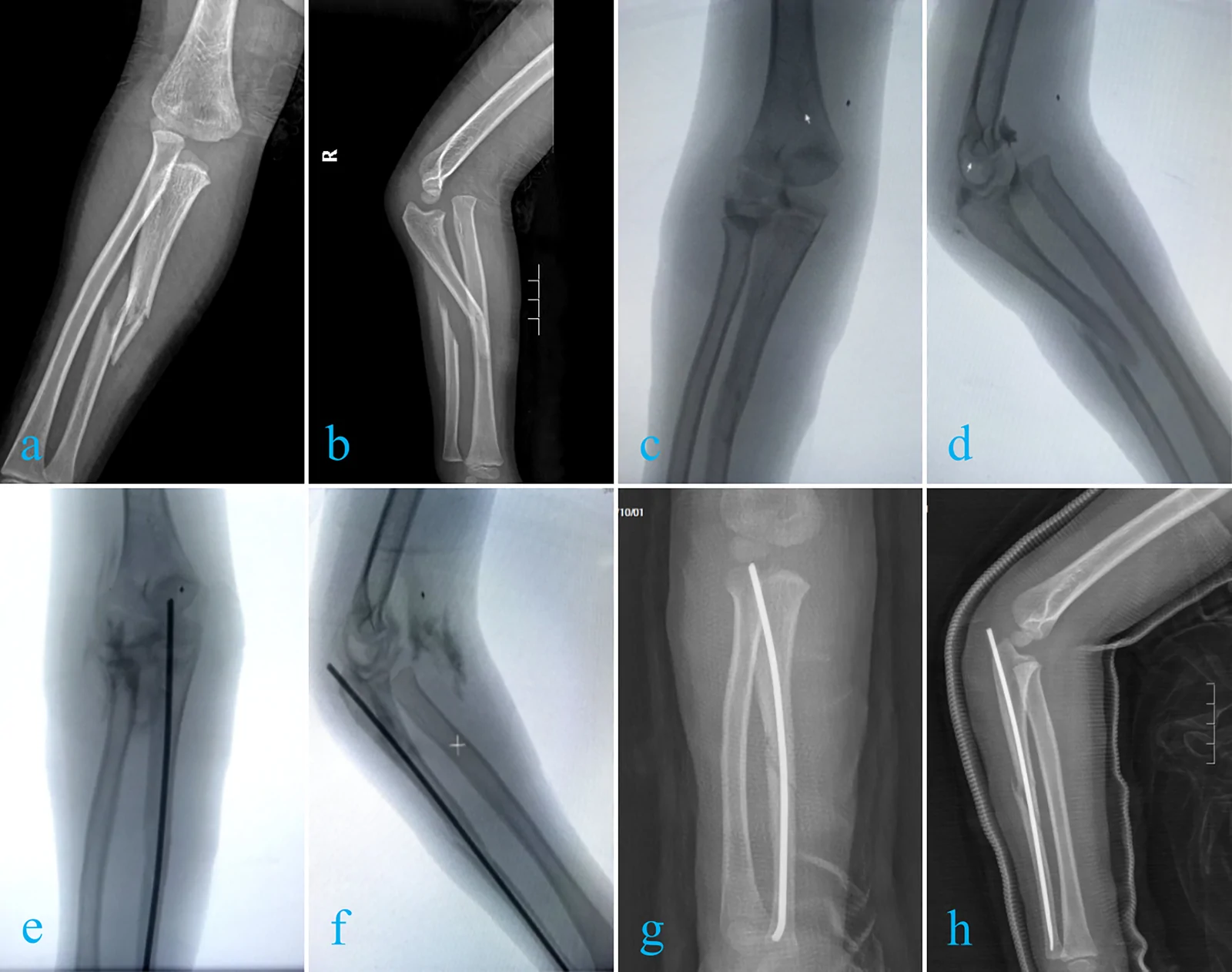
A 6-year-old girl with Type I Monteggia fracture. (a, b) Preoperative X-ray; (c–f) Treatment of Monteggia fracture under arthrography; (g, h) X-ray on the first day after surgery.

**Figure 2:**
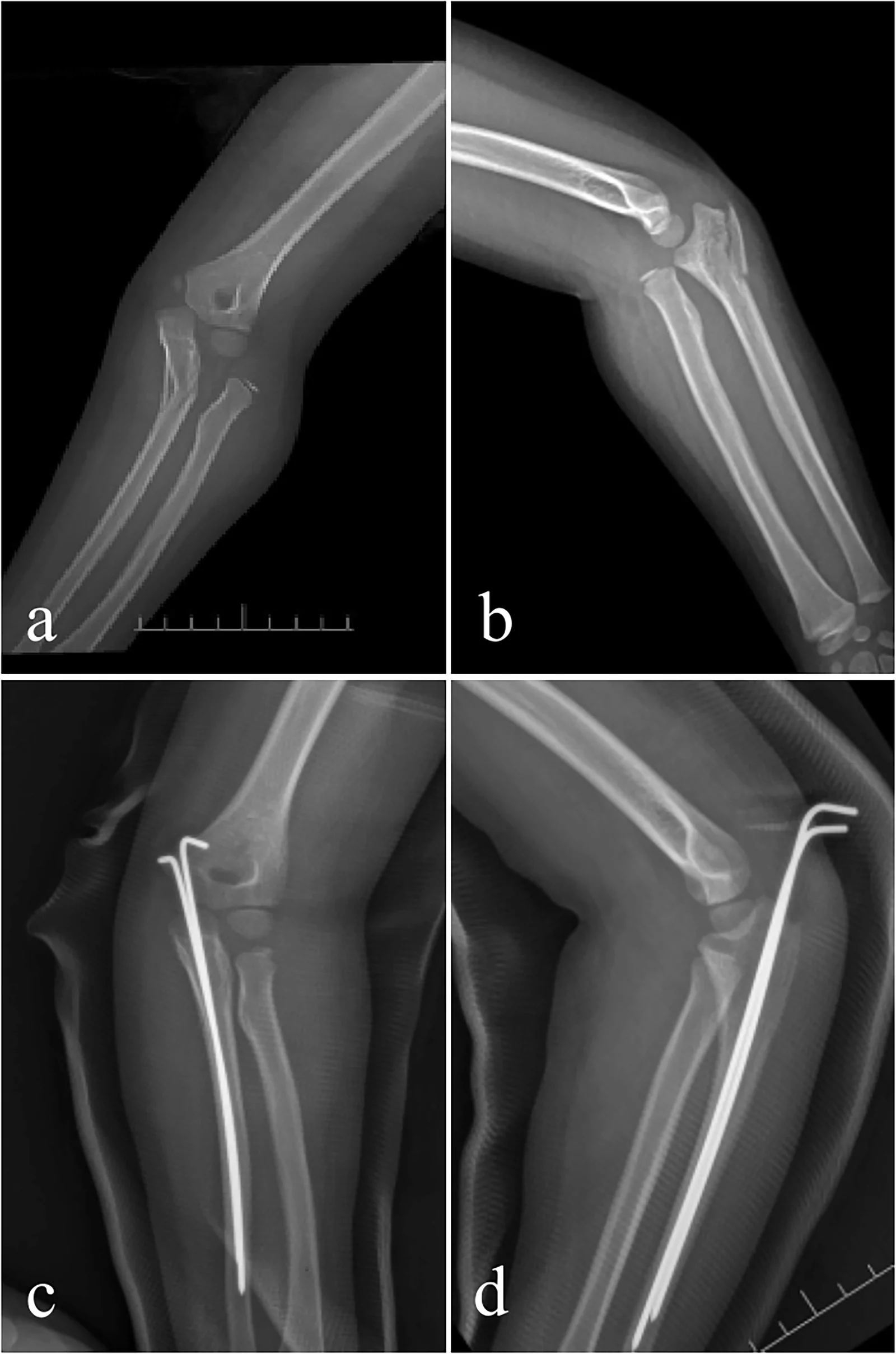
A 6-year-old girl with Type III Monteggia fracture. (a, b) Preoperative X-ray; (c, d) X-ray on the first day after surgery.

**Figure 3:**
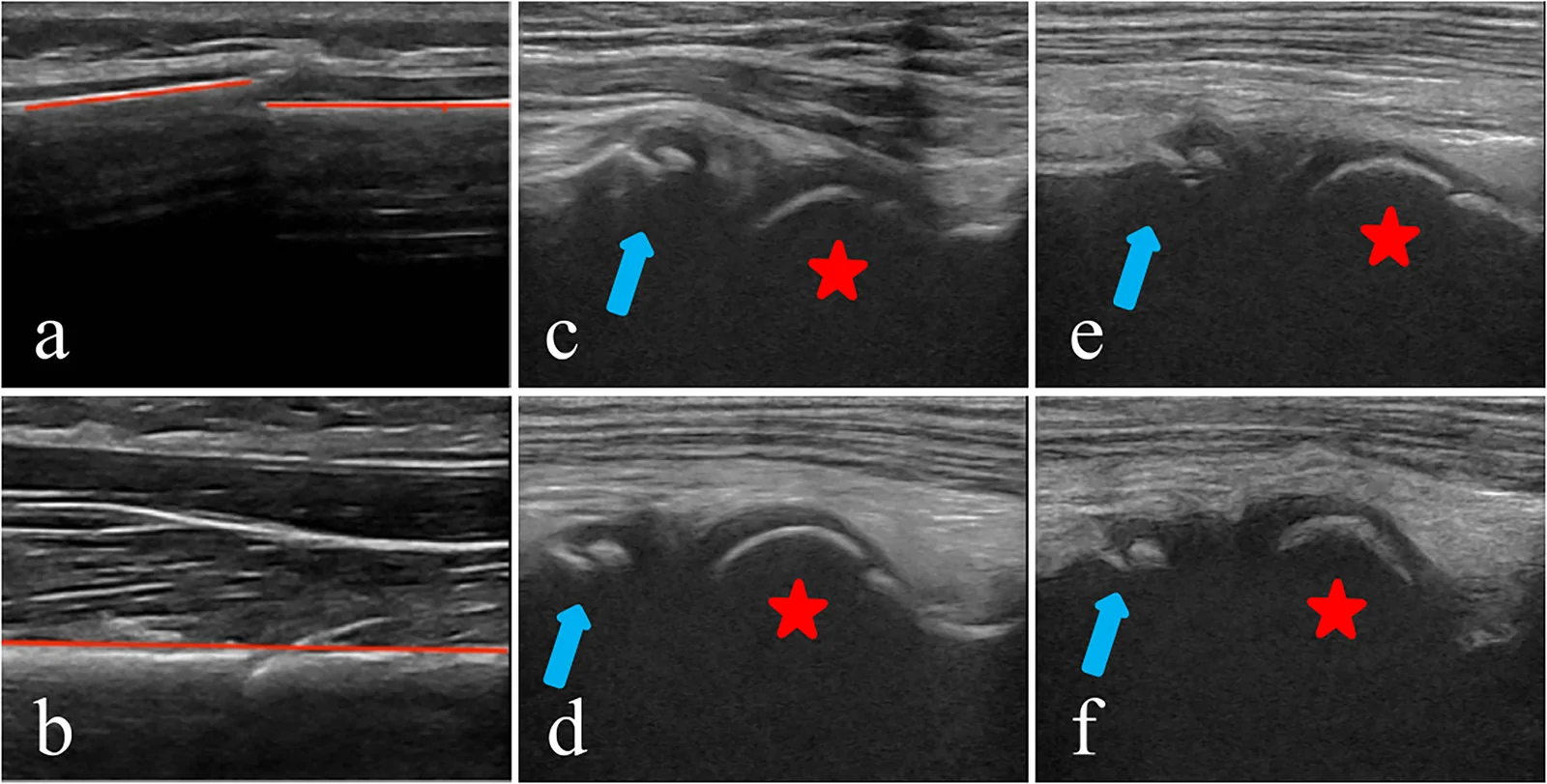
Treatment of Monteggia fracture under ultrasonographic guidance. (a, b) Reduction of ulna fracture (ulna fracture-red line); (c, d) Correction of anterior dislocation of radial head; (e, f) Correction of lateral dislocation of radial head (blue arrow-radial head, asterisk-Capitulum humeri).

## Postoperative management

Both groups received consistent postoperative management, with intraoperative fluoroscopy time, surgical duration, and internal fixation methods recorded separately. After surgery, plaster casting was applied for 4–6 weeks, followed by functional exercises for elbow flexion-extension and forearm rotation, along with elbow joint functional exercises upon plaster casting removal. The quality of reduction was assessed based on postoperative X-ray examination. The criteria for reduction evaluation were as follows: Excellent, no lateral displacement or displacement <1/4, no angular deviation or angular deviation <5°; Good, lateral displacement of 1/4–2/3, angular deviation of 5°–10°; Poor, lateral displacement >2/3, angular deviation >10° [8]. Complications such as recurrent radial head dislocation, fracture nonunion, pin tract infection, and neurological recovery were documented. The Mayo Elbow Performance Index was used to evaluate elbow joint function on the affected side during the last follow-up and compared with the flexion, extension, and rotation angles of the healthy side.

### Statistical analysis

SPSS 26.0 (Version 26.0; IBM, Armonk, NY, USA) was utilized for statistical analysis. Quantitative data were presented as mean ± standard deviation (SD), and we compared groups using an independent-samples *t*-test. Count variables were analyzed by chi-square or Fisher’s exact test and expressed as numbers. *P*-value <0.05 was considered statistically significant.

## Results

No significant differences were found in age, fracture side and type, associated injuries, time from injury to surgery, or internal fixation devices between children in Group A and those in Group B. Fluoroscopy time and surgical duration were significantly lower in Group B than in Group A (*P* < 0.05). One day postoperatively, we performed an X-ray examination. According to the reduction assessment criteria, Group A had 17 excellent cases and 2 good cases, while Group B had 20 excellent cases and 1 good case. All children were followed up for an average of 31.73 ± 16.85 months (range, 4–61 months). The Kirschner wires were removed 5–9 weeks after the surgery, and the ESINs were removed 4–10 months after the surgery. At the final follow-up, all fractures in both groups had healed, and all patients with radial nerve injuries had recovered well (Table 1). We assessed elbow joint function using the Mayo score: Group A had 18 excellent cases and 1 good case, and Group B had 20 excellent cases and 1 good case. In Group A, one patient developed residual anterior dislocation of the radial head postoperatively, which was corrected to achieve anatomical reduction through a secondary surgical revision. No complications such as pin tract infection or recurrent radial head dislocation occurred in Group B. The range of motion for elbow flexion, extension, pronation, and supination on the affected side showed no statistically significant difference between Group A and Group B (*P* > 0.05). (Table 2)

**Table 1:** Comparison of clinical data between two groups.

	Group A (*n* = 19)	Group B (*n* = 21)	Statistical value	*P*-value
Age (years) ($\bar{x}$ ± *s*)	5.26 ± 1.73	5.05 ± 1.43	*t* = 0.431	0.669
Sex			*χ* ^2^ = 0.422	0.516
Male	11	10		
Female	8	11		
Affected side			*χ* ^2^ = 0.819	0.366
Left	10	14		
Right	9	7		
Fixation devices			*χ* ^2^ = 0.852	0.356
Kirschner wire	9	13		
ESIN	10	8		
Bado classification			*χ* ^2^ = 0.961	0.811
I	8	9		
II	2	4		
III	7	7		
IV	2	1		
Time from injury to surgery (d) ($\bar{x}$ ± *s*)	2.32 ± 2.03	2.86 ± 2.99	*t* = 0.663	0.511
Fluoroscopy time ($\bar{x}$ ± *s*)	10.37 ± 3.48	2.62 ± 1.07	*t* = 9.307	<0.001
Surgical duration (min) ($\bar{x}$ ± *s*)	51.32 ± 18.47	36.67 ± 12.38	*t* = 2.972	0.005

**Table 2:** Comparison of the angles of flexion, extension, pronation, and supination between two groups at the last follow-up. ($\bar{x}$ ± *s*)

	Flexion (°)	Extension (°)	Pronation (°)	Supination (°)
Group A	140.21 ± 4.53	4.89 ± 2.96	86.16 ± 3.85	84.95 ± 2.76
Group B	142.20 ± 4.65	4.67 ± 2.78	86.86 ± 3.42	86.19 ± 2.79
*t* (value) *P* (value)	−1.361 0.182	0.251 0.803	−0.608 0.547	−1.416 0.165

## Discussion

Monteggia fracture is mostly caused by direct force combined with rotational force, resulting in a fracture of the proximal ulna with radial head dislocation [9]. It is commonly seen in children, and some cases are accompanied by radial nerve injury [10]. The treatment principle for Monteggia fracture in children is to restore and maintain the length and alignment of the ulna, while reducing the humeroradial joint [11]. If the fracture is not detected or treated in a timely manner, it may lead to malunion of the ulna and persistent radial head dislocation, resulting in an old Monteggia fracture. This, in turn, may cause elbow joint dysfunction, chronic pain, and degenerative arthritis [12, 13]. Most acute Monteggia fractures in children can be successfully treated with closed manual reduction and plaster casting. However, reduction failure and redislocation still occur frequently, and surgical treatment is often required for such cases [3, 14, 15]. Currently, assessing whether the radial head is properly reduced during surgery mainly relies on C-arm fluoroscopy of standard elbow joint anteroposterior and lateral views. In the humeroradial joint, the radial head and neck do not exceed the middle-outer one-third of the capitellum on the anteroposterior view, and the longitudinal axis extension line of the radial neck passes through the center of the capitellum on the lateral view. However, previous studies have shown that approximately 16% of children’s elbow joint development does not conform to these standards, which significantly increases the difficulty in assessing the humeroradial relationship and leads to missed diagnoses of radial head subluxation [16, 17].

Elbow arthrography allows for clear visualization of the contours of the capitellum and radial head under fluoroscopy by injecting a contrast agent into the joint cavity. This enables intuitive assessment of the humeroradial relationship and eliminates the interference of factors such as age and the presence of ossification nuclei [18, 19]. Especially in younger children, whose elbows are not fully ossified, intraoperative elbow arthrography can detect mild radial head subluxation that may not be evident on intraoperative fluoroscopy. Lee et al. [20] retrospectively analyzed preoperative X-rays and intraoperative arthrography results of 10 children with Monteggia fracture and found that the diagnosis in 5 children required further confirmation by intraoperative arthrography. If the articular surfaces of the capitellum and radial head are completely congruent and their edges are flush on both anteroposterior and lateral fluoroscopic views after arthrography, it indicates good reduction of the humeroradial joint. If the articular surfaces of the capitellum and radial head do not align or show notching at the edges, it indicates humeroradial dislocation or subluxation. Abnormal filling defects between the articular surfaces suggest soft-tissue impingement, such as the joint capsule or annular ligament, within the humeroradial joint. In our study, 19 children with acute Monteggia fracture underwent intraoperative elbow arthrography, resulting in good reduction of the radial head and functional recovery. However, if the dose of contrast agent is too small, the visualization of elbow structures may be poor. Conversely, excessive doses can easily lead to soft tissue leakage, causing blurring of elbow structures and making it difficult to accurately observe the congruence of the humeroradial joint. Additionally, arthrography still relies on repeated intraoperative fluoroscopy to monitor the quality of radial head reduction, inevitably leading to a substantial increase in radiation exposure for pediatric patients. Moreover, there is a risk of joint infection associated with joint puncture.

In recent years, ultrasound has gradually been applied as an intraoperative adjuvant therapy for treatment of pediatric elbow fractures and nerve exploration procedures [21–24]. By enabling real-time, multi-planar imaging of the cartilage-bone interface, it allows for intraoperative dynamic assessment of radial head concentric reduction and early detection of residual dislocation or redisplacement. In this study, 21 young children with acute Monteggia fracture underwent ultrasound-assisted treatment and achieved good reduction results. During the surgery, a sterile ultrasound probe was placed closely against the ulna; it can assist in accurately locating the entry point and angle for Kirschner wire placement, reducing the number of Kirschner wire insertions and subsequently avoiding secondary injuries caused by internal fixation implantation [22]. Furthermore, ultrasound can observe the congruence of the humeroradial joint through multiple planes and assess the degree of radial head reduction, eliminating the need for multiple intraoperative fluoroscopic verifications. It can also identify soft tissue entrapment within the humeroradial joint in cases of difficult or failed closed reduction, prompting timely conversion to open reduction and minimizing iatrogenic trauma caused by repeated reduction attempts. Additionally, for patients with preoperative radial nerve injury, ultrasound allows for intraoperative assessment of nerve continuity and morphological integrity [21]. Compared with elbow arthrography, ultrasound significantly shortened surgical duration, decreased surgical and anesthetic risks, and greatly reduced intraoperative radiation exposure. Studies have found that repeated intraoperative fluoroscopy leads to radiation exposure associated with an increased risk of solid organ tumor development and cataract formation [25]. Ultrasound is a non-invasive procedure that avoids the possibility of intra-articular infection caused by puncture. Given its high portability and applicability across operating rooms, outpatient clinics, and wards, ultrasound represents a practical and efficient adjunctive tool for pediatric Monteggia fracture management.

## Conclusion

In summary, for acute Monteggia fracture in young children, both elbow arthrography and ultrasound-assisted treatment can achieve satisfactory reduction results, with good postoperative recovery of elbow joint function. However, ultrasound not only shortens the surgical time but also significantly reduces the number of fluoroscopic exposures, thereby minimizing the potential risk of radiation-induced damage to children, surgeons, and other hospital staff.

**Abbreviation**
ESIN: Elastic stable intramedullary nail

## Data Availability

Upon reasonable request, the corresponding authors are committed to providing the original data to substantiate the conclusions presented in this paper.
